# Unique Exercise Lactate Profile in Muscle Phosphofructokinase Deficiency (Tarui Disease); Difference Compared with McArdle Disease

**DOI:** 10.3389/fneur.2016.00082

**Published:** 2016-05-30

**Authors:** Päivi Piirilä, Minna E. Similä, Johanna Palmio, Tomi Wuorimaa, Emil Ylikallio, Satu Sandell, Petri Haapalahti, Lasse Uotila, Henna Tyynismaa, Bjarne Udd, Mari Auranen

**Affiliations:** ^1^Unit of Clinical Physiology, HUS Medical Imaging Center, Helsinki University Hospital and University of Helsinki, Helsinki, Finland; ^2^Department of Clinical Nutrition Therapy, Helsinki University Central Hospital, Helsinki, Finland; ^3^Neuromuscular Research Center, Tampere University Hospital, University of Tampere, Tampere, Finland; ^4^Research Programs Unit, Molecular Neurology, Biomedicum Helsinki, University of Helsinki, Helsinki, Finland; ^5^Department of Neurology, Seinäjoki Central Hospital, Seinäjoki, Finland; ^6^Department of Neurology, Tampere University Hospital, Tampere University, Tampere, Finland; ^7^Laboratory of Clinical Chemistry, HUSLAB, Helsinki University Hospital, Helsinki, Finland; ^8^Department of Medical Genetics, Haartman Institute, University of Helsinki, Helsinki, Finland; ^9^Clinical Neurosciences, Neurology, Helsinki University Hospital, University of Helsinki, Helsinki, Finland

**Keywords:** ammonia, McArdle disease, lactate, muscle phosphofructokinase, pentose phosphate pathway, spiroergometry, Tarui disease, muscle metabolism

## Abstract

**Introduction:**

Glycogen storage disease V (GSDV, McArdle disease) and GSDVII (Tarui disease) are the most common of the rare disorders of glycogen metabolism. Both are associated with low lactate levels on exercise. Our aim was to find out whether lactate response associated with exercise testing could distinguish between these disorders.

**Methods:**

Two siblings with Tarui disease, two patients with McArdle disease and eight healthy controls were tested on spiroergometric exercise tests with follow-up of venous lactate and ammonia.

**Results:**

A late increase of lactate about three times the basal level was seen 10–30 min after exercise in patients with Tarui disease being higher than in McArdle disease and lower than in the controls. Ammonia was increased in Tarui disease.

**Discussion:**

Our results suggest that follow-up of lactate associated with exercise testing can be utilized in diagnostics to distinguish between different GSD diseases.

## Introduction

Tarui disease or glycogen storage disease VII (GSDVII) and McArdle disease (GSDV) are characterized by exercise intolerance, cramps and myoglobinuria or rhabdomyolysis, and very low lactate levels during exercise (1–5). In Tarui disease, reduced enzyme activity of muscle phosphofructokinase (PFKM) is detected resulting in impaired phosphorylation of fructose 6-phosphate to fructose 1,6-bisphosphate. In McArdle disease, a more proximal defect in the glycolysis chain is present based on the defect of glycolytic enzyme myophosphorylase.

We studied the lactate and ammonia profiles in two siblings with Tarui disease associated with symptom-limited maximal spiroergometric exercise testing. For comparison, two patients with McArdle disease and eight controls were studied. In defects of muscle metabolism, the level of lactate and/or ammonia associated with exercise may be altered depending on the character and location of the metabolic defect in the energy chain (5, 6). We report here the differences found in exercise-induced metabolites in these different study groups.

## Materials and Methods

### Patients and Controls

Patient Tarui 1 was a 58-year-old otherwise healthy man without regular medication. After age of 12 years, he began to experience strong attacks of muscle pain, weakness, cramping, and vomiting during extensive physical activity associated with increased CK levels. Mild muscle weakness was observed concentrating on hip flexors and extensors on both sides and ankle flexors and extensors on right side. No muscle atrophy was evident.

Patient Tarui 2, the 57-year-old younger sister of patient Tarui 1 had similar symptoms as her brother since the age of 10.

Both patients with Tarui disease showed in muscle electron microscopic analysis extra lysosomal glycogen accumulations. Although phosphofructokinase staining was normal, the whole-exome sequencing revealed a causative homozygous *PFKM* gene defect, R39Q, in both siblings establishing the diagnosis of GSDVII. Additionally, in biochemical studies, phosphofructokinase activity was reduced to 3–4% of normal activity in muscle tissue. A closer description of the biochemical, genetic, histological, and clinical findings of the patients has been reported previously (7).

For comparison, two male patients with McArdle disease, aged 35 (McArdle 1) and 20 years (McArdle 2) were studied. In both patients with McArdle disease, clinical symptoms, spiroergometric findings, and muscle biopsy analysis showing non-lysosomal glycogen accumulations and a total lack of myophosphorylase staining were consistent with McArdle disease. In line, a homozygous mutation of *PYGM* gene was detected in both of them (c.2056G > A; p.G686R in McArdle 1 and c.1A > G in McArdle 2).

For comparative analyses, eight healthy gender and age-matched control subjects were studied, the anthropometric characteristics are given in Table 1.

**Table 1:** **The main results of spiroergometric exercise testing as well as the venous lactate and ammonia results associated with exercise testing in patients with Tarui disease (Tarui 1 and Tarui 2), patients with McArdle disease (McArdle 1 and 2) and the controls**.

	Tarui 1	Tarui 2	McArdle 1	McArdle 2	Controls, *N* = 8, mean (SD)

Gender (m/f)	M	F	M	M	M/F 6/2
Age (y)	58	59	35	20	42.1 (15.9)
Height (cm)	177	155	174	163	176.4 (10.4)
Weight (kg)	71	76	64	57	74.9 (11.4)
Heart rate maximum (1/s)	173	155	182	176	176.0 (14.3)
Heart rate maximum percent of predicted^a^ (%)	98.3	88.3	96.8	90	92.6 (11.5)
Borg subjective scale 6–20	19	17	20	19	18.4 (1.3)
Breathing frequency (1/min)	68	29	34	30	32.0 (12.0)
RQ max	0.96	0.96	0.66	0.78	1.13 (0.07)
Wmax/3 min (maximal working capacity) (W)	93	50	50	80	246.5 (102.2)
Wmax/3 min percent or predicted^b^ (%)	56	37	21.8	38	115.9 (30.8)
V’O_2_max (maximal oxygen uptake) (L/min)	2.05	1.13	1.28	1.36	3.3 (1.36)
V’O_2_max% of predicted^c^ (%)	84.6	64.6	43	42	121.13 (37.2)
V’O_2_/kgmax (maximal oxygen uptake/weight) (ml/min/kg)	28.8	14.9	20	23.9	43.5 (14.3)
V’O_2_/kg max% of predicted^c^ (%)	91	62	51	52	121.5 (30.2)
Wmax/V’O_2_max (%)	13.1	12.7	11.2	13.3	21.2 (1.3)
Lactate at rest (mmol/l)	1.1	1.3	0.8	1.1	1.3 (0.45)
Maximal lactate (mmol/l)	3.2	2.4	0.9	1	11.7 (3.3)
Ammonia rest (μmol/l)	36	32	14	54	20.4 (11.0)
Maximal ammonia (μmol/l)	409	185	75	243	79.3 (33.7)

*RQ, (V’CO_2_/V’O_2_) respiratory quotient; V’O_2_max, maximal oxygen uptake; V’O_2_maxkg, maximal oxygen uptake per body weight; Wmax/V’O_2_max, mechanical efficiency*.

*^a^205 − 0.5 × age*.

*^b^Nordesjö and Landelius (8)*.

*^c^Seliger et al. (9)*.

Informed consent was signed by the patients and controls and the study has been performed in accordance with the ethical standards laid down in the 1964 Declaration of Helsinki and its later amendments (The Medical Ethics Committee of Helsinki and Uusimaa Hospital District, Finland; 3.8.2011, 199/13/03/01/11).

### Spiroergometry

A work-conducted maximal spiroergometric testing, i.e., bicycle ergometric testing with collection and analysis of breathing gases breath by breath during exercise was performed (10). A cannula was inserted in the left cubital vein, and the blood specimen for venous ammonia and lactate were drawn at following points: rest, first exercise step, maximal exercise, and 2, 4, 6, 10, 20, and 30 min after exercise. The workload was adjusted on the basis of subjects, previous exercise habits and reported exercise performance: in patient Tarui 1, McArdle 2, and the female control, the test was started with 40 W workload with increase of 40 W in 3-min steps (40 W/3 min) in patient Tarui 2 and McArdle 1 20 W/2 min and in the male controls with 50 W/3 min steps. The maximal subjective level of at least 17/20 was attained in all participants. The parameters measured are presented in Table 1.

### Laboratory Specimen Associated with Exercise testing

The lactate and ammonia specimens were taken into fluoride oxalate and EDTA syringes, respectively, centrifuged and analyzed with a Cobas Integra 400 + analyzer (Roche Diagnostics, Mannheim, Germany), and lactate and ammonia ions were assayed by enzymatic methods using lactate dehydrogenase and glutamate dehydrogenase, respectively.

## Results

The spiroergometric results are presented in Table 1.

**Figure 1:**
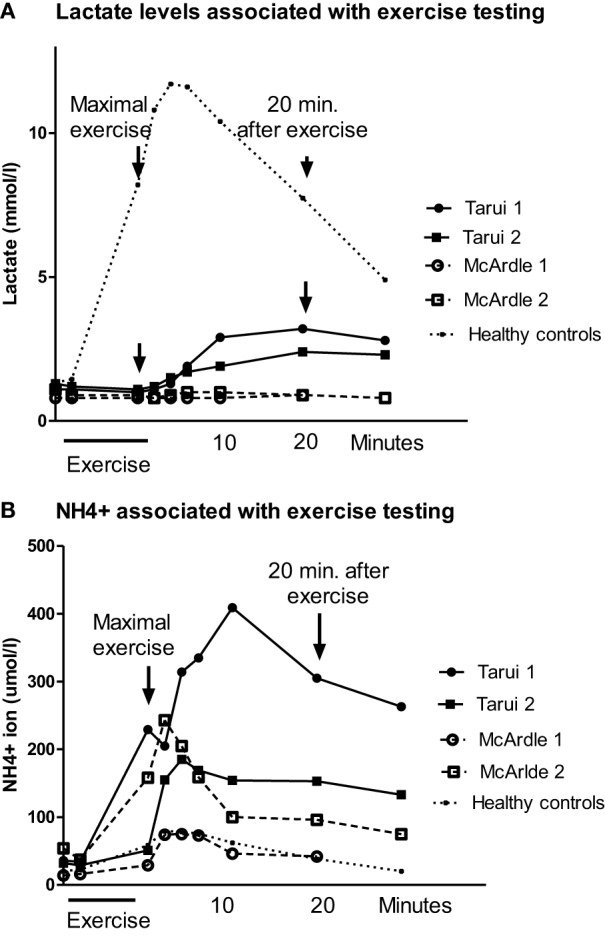
**(A)** The blood lactate levels associated with spiroergometric exercise testing in patients with Tarui disease and the results of two patients with McArdle disease are given, as well as the mean values of eight healthy controls matched to the age and gender of the patients. The blood samples were taken at rest, light exercise, maximal exercise, and 2, 4, 6, 10, 20, and 30 min after exercise. **(B)** The blood ammonia (NH_4_^+^) levels associated with spiroergometric exercise testing in patients with Tarui disease. For comparison, the results of two patients with McArdle disease are given, as well as the mean values of eight control subjects matched to the age and gender of the patients. The blood samples were taken at rest, light exercise, maximal exercise, and 2, 4, 6, 10, 20, and 30 min after exercise.

In patient Tarui 1, at maximum exercise a subjective strain of 19/20 was reached but the maximal working capacity for last 3 min (Wmax/3 min) was decreased (93 W, 56% of predicted). Although the breathing frequency was high (68/min) the respiratory quotient (RQ) (V’CO_2_/V’O_2_) remained below 1 (0.96). The oxygen uptake was normal but the mechanical efficiency (Wmax/V’O_2_ max) was reduced (13.1%). The venous lactate was at rest normal and remained at basic levels during the exercise but began to increase about 10 min after exercise with maximum at 20 min (Figure 1A). The ammonia level was normal at rest but exceptionally high after exercise (about 400 μmol/l) (Figure 1B).

Patient Tarui 2 showed also reduced exercise performance (Wmax/3 min 50 W, 37% of predicted) and oxygen uptake (14.9 ml/min/kg, 62% of predicted) (Table 1). Her RQ (V’CO_2_/V’O_2_) was similar as in her brother (0.96). Her mechanical efficiency was also reduced (12.7%). She showed a similar lactate response as patient Tarui 1, with a delayed increase beginning 10 min after exercise with a maximum at 20 min after exercise (Figure 1A). Also her ammonia level associated with exercise testing was higher than that in healthy controls (Figure 1B).

In the patients with McArdle disease, the maximal exercise capacity (22% of predicted values in patient McArdle 1 and 38% in patient McArdle 2), maximal oxygen uptake (20 and 24 ml/min/kg; 51 and 52% of predicted values, respectively), and mechanical efficiency (11.2 and 13.3%, respectively) were reduced. The RQ (V’CO_2_/V’O_2_) value remained lower than that in the patients with Tarui disease (0.66 in patient McArdle 1 and 0.78 in patient McArdle 2) and there were no signs of increased ventilation during exercise. The venous lactate level in the patients with McArdle disease remained low during the whole follow-up, and did not increase after exercise (Figure 1A). The ammonia level increased almost similarly as in the controls in patient McArdle 1 but was higher than in controls in patient McArdle 2 (Figure 1B).

The healthy controls had normal exercise performance and oxygen uptake (Table 1), and their RQ values at maximal exercise were clearly higher than 1. Their mechanical efficiencies were normal, with mean value of 21.2%.

The lactate and ammonia values of the control subjects increased normally with a maximal increase of lactate and ammonia 2–6 min after exercise.

## Discussion

In Tarui disease, the utilization of glycogen during anaerobic exercise is interrupted by the lack of PFKM leading to very low levels of lactate during exercise. However, in spiroergometric testing a late increase of lactate two to three times the basal value was seen at time points 10–30 min after exercise. As far as we know, this study demonstrates for the first time that exercise lactate profile distinguishes Tarui disease from McArdle disease.

At rest and during low exercise, adenosine triphosphate (ATP) molecules are generated through aerobic oxidative phosphorylation. Exercise requires increase of energy that can be attained by using anaerobic metabolism mainly through glycogen storages, which causes that lactate begins to accumulate in blood and elimination of lactate generates carbon dioxide (CO_2_) increasing minute ventilation and exhaled CO_2_ (V’CO_2_) related to oxygen uptake (V’O_2_) (11). Simultaneously, catabolism of nucleic acids as an energy source produces ammonia (12, 13).

Normally, glycogen is metabolized via glucose 6-phosphate to fructose 6-phosphate, but in Tarui disease fructose 1,6-bisphosphate cannot be produced. In our patients with Tarui disease, a slight residual PFKM activity of 3–4% out of normal was found in enzyme activity analyses, which might lead to a slight increase of lactate during exercise. However, if the residual phosphofructokinase production would increase the lactate level, the lactate raise should start during exercise with a maximum level of lactate 2–4 min after exercise, and not at time points 10–30 min exercise as we here demonstrate.

The late increase of lactate could arise a question whether the so called “second wind” phenomenon would be experienced by the patients Tarui 1 and 2. Second wind phenomenon occurs often in McArdle disease and is characterized by the patient’s better tolerance for aerobic exercise after a cumbersome initial exercise period with muscle pain and stiffness. Usually, second wind is explained by the muscle tissue adaptation, increased blood flow, and metabolic swift to utilize alternative sources of energy, such as free fatty acids (14, 15). Second wind phenomenon is not usually seen in patients with Tarui disease (16), although some opposing reports exist (17). In our patients, second wind phenomenon was observed only in patient McArdle 2. Therefore, it is not likely that the late increase of lactate in Tarui disease would be associated with second wind phenomenon.

**Figure 2:**
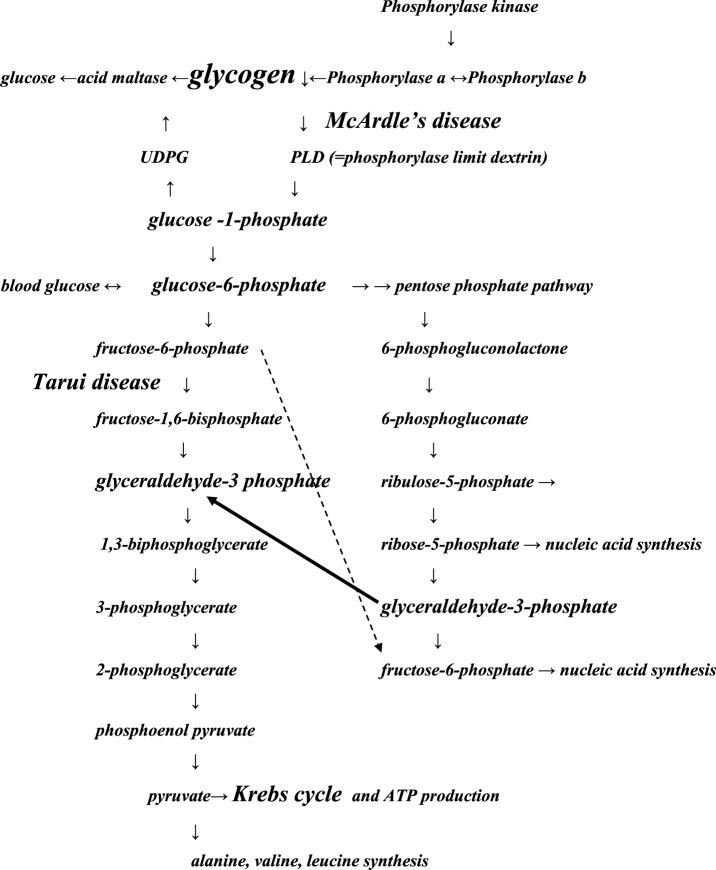
**Schematic presentation of the pathways of glycolysis modified from Ref. (18)**. The main points of the glycolysis chains involved in Tarui disease and McArdle disease are presented. The pathways may pass forward or backward. The black arrow indicates that glycolysis disrupted by Tarui disease can continue through pentose phosphate pathway (PPP) at glyceraldehyde-3 phosphate. The dotted arrow: fructose 6-phosphate accumulates because of Tarui disease, and its metabolism may continue in the PPP pathway, from where it may enter in protein or nucleic acid synthesis. Increase of synthesis of proteins or nucleotides means also increase of products of their metabolism, increasing, e.g., the production of ammonia. Some enzyme names and products of the glycolysis chain have been left away to get the figure more feasible to the present purpose. UDPG, uridine diphosphoglucose.

Our results suggest that in patients with Tarui disease an alternative route of glycogen metabolism is introduced. In the pentose phosphate pathway (PPP) (18) (Figure 2), the metabolism of glucose 6-phosphate continues to glyceraldehyde 3-phosphate, which may enter the glycolysis chain distally to the PFKM step leading to lactate production. The complicated route could probably explain the late increase of lactate beginning 10–20 min after exercise seen in Tarui disease compared to controls, in whom the maximal lactate increase was present 2–4 min after exercise. However, in McArdle disease, the defect of muscle phosphorylase disrupts the metabolism of glycogen at the beginning of the glycogenolytic cycle causing that no increase of lactate level occurs during or after exercise (4, 5).

In some previous studies on Tarui disease, suggestions of a late lactate increase after exercise have been recorded, confirming our results (19, 20). In addition, findings that suggest increased glyceraldehyde-3 phosphate levels and alteration into the PPP route in Tarui disease during exercise have been published (3). We report here for the first time a full-length spiroergometry study with follow-up of lactate and ammonia, as well as extended follow-up after exercise, comparing the results also with healthy controls.

During exercise, the patients with Tarui disease showed exceptionally high ammonia levels that has previously been explained by overuse of proteins because of decreased glycogen metabolism (21–23). We suggest that also the activation of PPP route and the raise in ribose 5-phosphate could boost the ammonia levels by influencing the amino-acid synthesis. The deamination of AMP, as well as of various amino acids and the metabolites of citric acid cycle generate ammonia (13, 24).

In McArdle disease, increased ammonia during exercise has previously been reported (4, 25–28), as also we found in patient McArdle 2. Compared to patient McArdle 2, patient McArdle 1 showed only slight increase of ammonia in exercise even though he reached maximal subjective level of 19/20. Earlier, Mineo et al. (19) have found corresponding slight ammonia responses in McArdle disease. Heterogeneity of McArdle disease might be one explanation for the observed variances in ammonia level. In addition, it is difficult to assess objectively the maximality of exercise in McArdle disease because the absent lactate response causes that ventilation is not stimulated normally during exercise, and also the RQ value remains low.

In conclusion, we show that Tarui disease is associated with low lactate levels during exercise with a late increase of lactate after exercise and exceptionally high ammonia levels during and after exercise. The particular lactate profile differentiates patients with Tarui disease from patients with McArdle disease and helps the clinician to choose proper genetic tests. As far as we know, this phenomenon has not reported earlier, and it suggests that further study should be performed on exercise glucose metabolism in patients with rare glycogen storage disorders. In diagnostic setting, we recommend to utilize maximal bicycle spiroergometry with sufficiently long (30–40 min) follow-up of ammonia and lactate levels after exercise.

## Author Contributions

PP: spiroergometric testing of patients Tarui 2, McArdle 1, and control subjects, interpretation and analysis of data, and drafting of the article; MS: treatment of the patients with Tarui disease, data analysis, and drafting of the article; JP: clinical study, data analysis, and drafting of the article; TW: spiroergometric testing of patient Tarui 1, spirorgometric testing of the control subjects, interpretation and analysis of data, and drafting of the article; EY: genetic testing, data analysis, and manuscript drafting; SS: clinical study of patients with Tarui disease, data analysis, and drafting of the article; PH: spiroergometric testing of patient McArdle 2, spiroergometric testing of the control subjects, and manuscript drafting; LU: the biochemical expert in the study, drafting and critical revision of intellectual biochemical contents of the data; HT: genetic studies and data analysis, and manuscript drafting; BU: clinical examinations, examinations of patient Tarui 2, data analysis, and drafting of the article; MA: clinical examination of patients Tarui1 and patients McArdle 1 and 2, data analysis, and drafting of the article.

## Conflict of Interest Statement

The authors declare that the research was conducted in the absence of any commercial or financial relationships that could be construed as a potential conflict of interest.
